# Physical and Chemical Methods for Reduction in Aflatoxin Content of Feed and Food

**DOI:** 10.3390/toxins13030204

**Published:** 2021-03-12

**Authors:** Péter Sipos, Ferenc Peles, Dóra Lili Brassó, Béla Béri, Tünde Pusztahelyi, István Pócsi, Zoltán Győri

**Affiliations:** 1 Institute of Nutrition, Faculty of Agricultural and Food Sciences and Environmental Management, University of Debrecen, Böszörményi str. 138, H-4032 Debrecen, Hungary; gyori.zoltan@unideb.hu; 2 Institute of Food Science, Faculty of Agricultural and Food Sciences and Environmental Management, University of Debrecen, Böszörményi str. 138, H-4032 Debrecen, Hungary; pelesf@agr.unideb.hu; 3 Department of Animal Husbandry, Faculty of Agricultural and Food Sciences and Environmental Management, University of Debrecen, Böszörményi str. 138, H-4032 Debrecen, Hungary; brasso.dora@agr.unideb.hu (D.L.B.); beri@agr.unideb.hu (B.B.); 4 Central Laboratory of Agricultural and Food Products, Faculty of Agricultural and Food Sciences and Environmental Management, University of Debrecen, Böszörményi str. 138, H-4032 Debrecen, Hungary; pusztahelyi@agr.unideb.hu; 5 Department of Molecular Biotechnology and Microbiology, Institute of Biotechnology, Faculty of Science and Technology, University of Debrecen, Egyetem Square 1, H-4032 Debrecen, Hungary; pocsi.istvan@science.unideb.hu

**Keywords:** aflatoxin, mycotoxin, feed chain, food chain, decontamination, absorption

## Abstract

Aflatoxins (AFs) are among the most harmful fungal secondary metabolites imposing serious health risks on both household animals and humans. The more frequent occurrence of aflatoxins in the feed and food chain is clearly foreseeable as a consequence of the extreme weather conditions recorded most recently worldwide. Furthermore, production parameters, such as unadjusted variety use and improper cultural practices, can also increase the incidence of contamination. In current aflatoxin control measures, emphasis is put on prevention including a plethora of pre-harvest methods, introduced to control *Aspergillus* infestations and to avoid the deleterious effects of aflatoxins on public health. Nevertheless, the continuous evaluation and improvement of post-harvest methods to combat these hazardous secondary metabolites are also required. Already in-use and emerging physical methods, such as pulsed electric fields and other nonthermal treatments as well as interventions with chemical agents such as acids, enzymes, gases, and absorbents in animal husbandry have been demonstrated as effective in reducing mycotoxins in feed and food. Although most of them have no disadvantageous effect either on nutritional properties or food safety, further research is needed to ensure the expected efficacy. Nevertheless, we can envisage the rapid spread of these easy-to-use, cost-effective, and safe post-harvest tools during storage and food processing.

## 1. Introduction

Mycotoxins are widely known deleterious secondary metabolites produced by various molds. The furanocoumarin derivative aflatoxins (AFs) are among the most significant and most harmful mycotoxins contaminating feed and food and, as a consequence, imposing real threats on the health of both domestic animals and humans initiating various highly pathological cellular and physiological processes [1,2]. The mutagenic, teratogenic, genotoxic, and carcinogenic effects of AFs have also been confirmed, primarily when the consequences of long-term exposures to them are evaluated [1,3]. Expectedly, dysfunctions of many organs of AF-exposed humans and animals have been reported, including the liver, kidneys, the gastrointestinal tract, and the reproductive and immune systems [1]. Additionally, AFs may disturb the early, even embryonic, development of humans, resulting in growth and mental retardations and immune system dysfunctions [2].

AFs are produced by several *Aspergillus* species [4], among which *Aspergillus flavus*, *A. parasiticus*, *A. nomius*, and *A. pseudotamarii* are regarded as primary AF producers [2,4,5]. The elements and regulations of the gene clusters responsible for AF production in the *Aspergilli* are also well known and are still intensively studied [1,6]. Oxidative stress is likely to play a pivotal role in the activation of the AF gene cluster [6,7,8]; however, the ecology of toxin production of *Aspergilli* is remarkably complex. The multilevel interactions of AF producer fungi, plant hosts, and soil micro- and macrobiota should be studied in depth to make the development and improvement of AF prevention strategies more effective [9]. Several pre-harvest biological control methods have been shown to be effective to mitigate the toxic effects of AFs, which are based on the advantageous, competitive-exclusion, or biofungicide characteristics of different bacteria, yeasts, fungi, and their excretes, which has already led to the successful development and implementation of a number of combined pre-harvest biocontrol technologies [10].

Hazardous AF contaminations can occur at any point of the feed and food chain starting from field production to the final use of a wide variety of plant products, such as cereals, nuts, spices, and fruits [5,11,12,13,14]. AFs are transferred into different body parts of animals and humans after consumption and absorption from the gut, and they can even be modified chemically, giving rise to an array of further dangerous derivatives. These harmful compounds such as AFM1 will be eventually excreted and appear even in milk [15]. Dangerous indirect AFM1 contaminations of milk and dairy products have been reported in the literature in outstandingly high numbers, and the direct AF contaminations of milk products by molds and their mycotoxins have also been published [16,17,18,19]. To make matters even worse, AFs may also appear in human breast milk after conversion to AFM1, which is definitely threatening for highly susceptible breastfeeding newborns [20,21]. AFs also provoke developmental disorders of embryos in utero after passing through the placenta [22].

Recent research performed in the development and improvement of AF control technologies focuses on both prevention and good storage and manufacturing practices that can be applied in the feed and food chain to reduce AFs exposure, but these efforts are not always satisfactory to ensure food safety [3]. Therefore, recent research activities seem to be shifted towards reducing the AF contents already present in feeds and foods, and several biological, physical, and chemical methods have been tested and evaluated in the mitigation of AFs in this way (Table 1). Biological detoxification methods rely on specific microorganisms, which bind and/or transform AFs into less toxic compounds [23,24] and which are also advantageous in terms of the sensory and nutritional values of food and represent a safer option to choose considering food safety aspects [10]. However, due to the inherent nature of these methods, they typically cannot be applied for a wide spectrum of commodities. Physical and chemical methods can also be applied safely and with high efficiency, and they are generally much faster than the biological methods, which makes them much more acceptable for potential consumers [25]. Traditional and novel technologies and practices for AF mitigation in feed and food are summarized and discussed in this review, paying a special attention to the physical (sorting, dehulling, heating, irradiation, and cold plasma treatment) and chemical (e.g., acidification, ozonation) detoxification methods available in feed and food processing with an emphasis on the most promising novel and innovative approaches and technologies.

## 2. Reduction in Aflatoxins in Feed and Food

The post-harvest methods employed to decrease the AF contents of foraging can be classified into three main groups: physical, chemical, and biological ones [26,27]. However, there is another way to classify the AF-reducing technologies, which distinguishes natural, physical, and chemical methods. The natural methods cover all those physical applications such as sorting, cleaning, and screening, where AFs are not destructed, inactivated, or absorbed. Instead, those particles, which show signs of possible contamination, including detectable differences in size, color, and/or shape, are removed from the seed lots by various physical approaches [5].

### 2.1. Physical Methods

The post-harvest physical processing operations have been widely evaluated (Table 2), and several recommended measures have been found useful in reducing AF levels. For example, hand sorting by visible fungi infection is found to be a very efficient tool to decrease the AFB1 concentration of corn kernels. Nevertheless, this approach is only applicable on an industrial scale using optical sorting equipment [28]. When feed corn grains were sorted visually into three groups based on the content of foreign materials and moldy and damaged grains, the aflatoxin concentration increased from the best graded to worst graded [29]. On the other hand, size separation, e.g., sieving, can also be useful, as the small components such as broken kernels may be infected or damaged by fungi and can be a source for further spoilage [30]. Size separation of in-shell Brazil nuts was also found in toxin reduction, only the small ones contained AFB1 [31]. There are quantifiable differences in the major and minor diameters, sphericities, densities of *Aspergillus* contaminated and healthy corn kernels and industrial use of screen cleaner and gravity table resulted in significantly decreased AF contents [32].

Dehulling is also an effective physical tool for fungal and mycotoxin decontamination of grains where it can be applied [30]. For example, it can remove more than 90% of the original AF content from the corn kernel [37]. Several other researchers reported that removing the external layers of the kernel decreased the AF content of grains significantly, and the efficiency of this application can be much more remarkable by floating and washing [28,33,34]. Rice kernel polishing is also a recommendable process for AF reduction resulting in more than nine-fold decreases in mycotoxin content [38].

AFs are highly resistant to heat treatment, since their decomposing temperature is higher than 235 °C [30,68,70]; therefore, simple drying cannot decrease their concentrations in stored grains significantly. However, long-time high-temperature treatments seem to have a beneficial effect on decontamination: 100 and 150 °C heat treatments for 90 min resulted in significant decreases (41.9 and 81.2%, respectively) in the AFB1 contents of soybean [41]. The application of higher drying temperature for a longer time decreases both infection and toxin content. Similar degradation ratios were observed during dry heat treatment of wheat grains [40]. Roasting between 90 and 150 °C for 30 to 120 min reduced AF concentrations in peanuts and pistachio by 57–90 and 93%, respectively [42,43]. The cooking of maize can decrease AF content by 51 to 85% [44]. Simple rice cooking also yielded a 34% decrease in AFB1 content [45], which could be improved up to 88% using high pressure [46]. Extrusion of cornmeal decreases AFBs content by 80.5 to 83.7% and AFGs content by 74.7 to 87.1%, and with the addition of amylose-rich corn starch, the effectiveness of reduction increased by 0.3 to 8.1% [47]. Instant catapult steam explosion, initially used for the deconstruction of lignocellulose biomass, was found similarly effective combined with heat–pressure treatment. The AFB1 content of treated corn stalk yielded 78.8 to 100.0% decrease; the efficiency of treatment is higher by increasing the applied pressure and temperature, resulting in a toxin-free feed owing to the combined effects of hydrolysis–oxidation–dehydrogenation and dehydrogenation–decarbonylation pathways [48]. According to previous reports, moisture content, heating temperature, processing time, and the properties of the food matrix are the critical efficiency parameters in thermal unit operations [40,41,42,43,46,47,48].

Due to the adverse effects of heat treatments on nutritional properties, the food industry is increasingly interested in non-thermal technologies. For example, high pressure techniques are ecologically friendly treatments that do not affect significantly either the nutritional status or the organoleptic properties of foods; meanwhile, they significantly reduce spoilages caused by microorganisms and enzymes [71]. High-pressure (500 MPa) treatment for 5 min was successfully applied to decrease the AFB1, B2, G1, and G2 contents of spiked water samples by 61–87%; however, the degradation aflatoxins was significantly less effective in artificially contaminated grape juice samples (14–29%) [49].

Another physical method, which can be employed for the reduction in AF contamination, is irradiation. When *A. flavus* contaminated corn kernels are illuminated with UV light, they emit bright greenish-yellowish light making separation possible. However, this reaction is not visible in every case, and the internal fungal contaminations have no visible effects either [72]. An AF-containing peanut sorting method based on red and green light reflectance was also developed [73]. A low-cost multi-spectral analyzer was developed to monitor single corn kernels at nine distinct wavelengths in the λ = 470–1550 nm region for qualitative use [36], which can also be applied in the cleaning process. The fluorescent technique showed higher sensitivity and specificity than near-infrared spectroscopy and hyperspectral imaging; however, near-infrared spectroscopic evaluation has high capability on both AF and fungal contamination and has already been applied in automatic sorters in practice [74].

Pulsed electric field (PEF) is a relatively new processing method, and it takes advantage of short pulses of electric field from 80 kV/cm to 100 V/cm, which alters the permeability of cell membranes. While PEF can be applied to improve material transfer processes, increasing the strength of the electric field will elicit antimicrobial inactivation and AF degradation processes. PEF treatments can decrease both AFB2 and AFG1 contents of contaminated water and grape juice samples; however, the efficiency of the method was dependent on the food matrix [50] and increased with increasing exposure periods [74]. Pulsed high-power ultrasound radiation was also effective and resulted in cavitation in the treated material, significant structural changes and inactivation of proteins and enzymes [75]. In another study, pulsed ultrasound treatment at 1.65 W/cm^3^ power intensity for 10 min reduced the AFB1 content of maize flour slurry with 11% removal rate [63].

AFs are destroyed by UV light in the presence of oxygen. The absorption maximums for various AFs are different: AFB1 shows maximum absorption at 223, 265, and 362 nm, AFB2 at 265 and 363 nm, AFG1 at 243, 257, 264, and 362 nm; meanwhile, AFG2 has its absorption maxima at 265 and 363 nm [76]. When AFG1, AFB1, and AFB2 were spiked in pure water and were treated by UV light, significant decreases in their concentrations (67.22, 98.25, and 29.77% decreases, respectively) were recorded [77]. Higher dose and more extended UV-C treatment (6.18 kJ/cm^2^, 3 h) decrease the AFB1 content of brown, black, and red rice [51]. The key issue of the efficiency of UV treatment is that the radiation has to be applied on the whole surface ensuring uniformity: e.g., rotation of peanuts during UV-C treatment resulted in 25% higher AFB1 degradation [52].

Gamma irradiation reduces both the number of fungi and the AFB1 content of naturally contaminated corn kernels: irradiation doses between 1 and 10 kGy can result in 69.8 and 94.5% mycotoxin reductions, respectively [55,56]. For corn, wheat, and rice kernels, 4, 6, and 8 kGy doses were found effective in 15–56% AF reductions by the increasing doses [54]. Higher than the 10 kGy dose was found to be useful in AFB1 reduction in soybean [57]. Lower values for AF decreases (20–43% decrease for 5–9 kGy doses) were reported in peanuts and found that low power microwave heating was much more effective (59–67% decrease at 360, 480, and 600 W), although the combination of the two irradiations resulted in a higher than 95% reduction [58]. When the effects of 5–25 kGy dose gamma irradiations on the AF content of artificially contaminated mixed poultry feed were examined, lower efficiency, 5–43% decrease in total aflatoxin and aflatoxin B1 content was found [53]. Meanwhile, the direct natural sunlight applied on the same media placed on a tray in 1–2 mm thickness for 30 h yielded higher decontamination ratios (up to 75%). It is noteworthy that three hours of sunlight treatments already had 40% efficiency under the same irradiation conditions [53]. Instead of direct light, pulsed light has also been evaluated and found to be an applicable technology for AF decontamination and was also found useful in the decontamination of solid materials [78]. Polychromatic light in the wavelength spectrum of 100–1100 nm applied with a xenon flash lamp resulted in 75 to 90% decreases in AFB1 and AFB2 contents of rice and rice bran [59]. Electric beam treatment was also evaluated in toxin mitigation of red pepper powder, and while it was useful in ochratoxin A reduction, it was ineffective in AFB1 degradation [79].

Cold (or non-thermal) plasma can also be used against fungal pathogens and their toxins. Cold plasma is a result of atmospheric dielectric discharge, which causes the ionized gas to contain metastable atoms and molecules with a nearly zero net electrical charge. The mycotoxin degradation is attributed to these free radicals (for example, O•- and OH•) [80]. The effects of pressure (atmospheric or vacuum), air composition, humidity, flow rate, discharging power, and treatment time on the efficiency of cold plasma application are under continuous evaluation nowadays [80]. A 30 min treatment of AFB1-spiked food samples at 1.5 A and 15 mm distance from the electrode can result in a 95% decrease in toxin concentration [67], and the efficiency of treatment is proved on naturally contaminated samples too. Cold plasma was more active on hazelnuts than a 10 kGy dose gamma irradiation and resulted in a 72–73% decrease in AF contents compared to the 47% efficiency of irradiation [65]. A 20 to 52% decrease in aflatoxin content was measured on different kinds of nuts [64,66,68], on corn [32], and wet and dried distilled grain with solubles [68]. Cold plasma is found as a cost-effective and ecologically friendly treatment, not significantly affecting the quality of kernels when compared to other detoxification methods [81]. This procedure can reach total detoxification of AFs, as it cleaves the vinyl bond between the 8 and 9 position on the terminal furan ring of AFB1, suppressing its toxic potential [82].

The use of electrolyzed water can result in significant degradation of the AF content of different substrates. For example, soaking of grains in electrolyzed acidic water for 15 min resulted in an 85 to 90% decrease in AB1 of peanuts [68]. The mechanism of inactivation caused by chlorenium and hydroxide ions [83]. In another study, alkaline-electrolyzed water was found useful in removing AFB1 from peanut and olive oil [69]. Carbon filtration can be also applied for AF removal from liquid materials, e.g., in coffee samples spiked with different concentrations of AF resulted in a 73 to 78% decrease, and therefore, it seems to be an effective and cheap strategy to AF control and mitigation [39].

Post-harvest losses in stored maize can be significant due to storage pests such as the maize weevil or the larger grain borer, improper storage conditions, and practices. Awuah et al. (2019) evaluated the effectiveness of packaging with triple-layer hermetic and standard woven polypropylene bags in Ghana. The triple layer of hermetic bags significantly decreased the growth of the pest populations, and AF counts were considerably worse in woven polypropylene packaging (16.39%) than in the triple-layer hermetic bags (3%) under both ambient and simulated hot storage conditions [84]. Another evaluation done in Nigeria revealed that Purdue Improved Crop Storage (PICS) hermetic bags were the most efficient in mitigating pest population growth [85]. The effectiveness of the treatments decreased in the order PICS > ZeroFly bags > polypropylene bag control. However, the purchasing power of and the willingness to buy such hermetic bags in Malawi’s very low-income farmer communities was low even among those who attended demonstrations [86].

### 2.2. Chemical Methods

Current chemical methods to be chosen for AF reduction are traditionally based on various chemical agents, ozonation, and adsorption; however, there are a number of emerging chemistry-based techniques as well (Table 3). Some organic and inorganic acids such as citric, lactic, tartaric, propionic, and hydrochloric acids seem to be more effective than others, such as succinic, acetic, ascorbic, and formic, which were found only marginally successful [87,88]. Citric acid treatment resulted in a remarkably high 86–92% decrease in the AFB1 content of duckling feed; meanwhile, only a more moderate 67% decrease was recorded with lactic acid solutions [89,90]. The AFB1 decomposing effect of sodium bisulfite, an effective reducing agent relies on the formation of sulfonate derivate [91], which can be significantly enhanced by concomitant heat treatment and the addition of ozone and hydrogen peroxide. Using this technique at 25 °C, a 28% mycotoxin reduction was achieved in AFB1-contaminated dried fig fruits, which efficiency was increased further up to 65% when 0.2% H_2_O_2_ was added 10 min before sodium-bisulfite treatment, and 48 and 68% AFB1 reduction was documented when 45 and 65 °C heat treatment was employed for 1 h after the addition of reducing agent [92]. The oxidizing agent ammonium persulfate was also with a 31 to 51% decrease in the AFB1 contents [93]. Aflatoxin-reducing effects of ammoniation were also reported in an alkaline environment [87].

Sodium hydrosulfite is also a useful chemical agent in AF reduction. A 96 to 100% decrease in the AF content of black pepper was found when sodium hydrosulfite was applied in concentrations selected in the range of 0.25 to 2% both under atmospheric and high pressures [94]. Several acidic and alkaline compounds and salts can also be used to decrease total AFs in white and black pepper [95]. The application of chloridric acid, phosphoric acid, sodium, potassium, calcium hydroxide, sodium bicarbonate, sodium bisulfite, sodium hydrosulfite, sodium chloride, and sodium sulfate resulted in a 18 to 51% reductions in AF concentrations; however, the addition of pure water alone also resulted in 13 to 20% shrinkages of the same AF pools [95].

Certain redox-active enzymes can be regarded as novel tools in chemistry-based AF mitigation procedures. For example, a recombinant type B dye-decolorizing peroxidase (Rh_DypB) was also effective in in vitro digestion of AFB1 [97]. Depending on the experimental setup, 96% of bioconversion was reached after 96 h by the addition of 0.1 U/mL enzyme and 0.1 mM H_2_O_2_, which is promising and can be applicable in mycotoxin mitigation in feeds.

Another novel chemical method is the employment of the antifungal and insect-killing ozone during grain storage to control AF [101]. The highly reactive O_3_ molecules may take part in the direct mycotoxin reduction as well without any adverse effects on food quality [103,104]. Ozone destructs AFs with high efficiency (up to 66–95% of the initial toxin concentration) in cereal grains and flours, soybean, and peanut [97,102,105].

A broad spectrum of chemicals can inactivate AFM1 in milk, and various ammoniation, acidification, oxidative, and reductive technologies based on them have been developed and tested. By applying ammoniation, the AFM1 content can be reduced by 79 to 90% [96]. The application of 0.5–2.0% ammonia under high pressure (45–50 psi) with 12–16% of moisture at 80–100 °C for less than an hour is considered the most effective method to reduce the AFM1 content of milk [106]. Chlorine dioxide gas is also an effective detoxifying agent to mitigate AFB1-contaminated maize [107].

Adsorption has also been evaluated as a possible mycotoxin-reducing method, and different kinds of adsorbents, nanoparticles, nanocomposites, and magnetic-activated carbon were tested extensively. These absorbents are suitable agents for AFB1 decontamination in poultry feed [108] and in AFB1-contaminated vegetable oils [109,110,111]. In these experiments, the dose of adsorbents, treatment time, temperature, initial toxin concentration, and pH were the critical parameters of efficiency [110,111]. The AF-reducing capability of chitosan nanoparticles has also demonstrated in several studies [112,113]. Considering that mycotoxin-binding absorbents are quite commonly used to alleviate AFs in animal, especially ruminant feed, they are presented in more detail in a separate section as shown below.

### 2.3. Agents Detoxifying AFs in Animal Husbandry

Mycotoxin detoxifying agents used in animal husbandry have been reviewed extensively in some recent publications [91,114,115,116]. AFB1 binders traditionally attract lots of interest from researchers as well as from agricultural experts and farmers [115,117,118]. Adsorbents with multi mycotoxin binding efficacy are also in the limelight of current research performed in this field [114,119,120].

Mycotoxins can enter into the livestock organism via adsorption. After intake, AFB1 transformation to AFM1 and AFM2 takes place in the liver and can have unfavorable effects on the organs [121]. To reduce the presence of mycotoxins in the gastrointestinal tract and prevent their further spread in the tissues, adsorbents are added to feeds or used separately at mealtime. There have been various mycotoxin binders tested and employed in the last years, such as minerals (bentonite, vermiculite, nontronite, montmorillonite, activated carbon, glucomannan, zeolite, hydrated sodium calcium aluminosilicate (HSCAS), sepiolite and diatomite), chemicals, organic adsorbents (yeast, lactobacilli, micronized fibers, and bio-sorbents), and as a long-known and long-practiced solution, synthetic polymers [122,123] (Table 4.).

One of the most widely used minerals is the aluminum phyllosilicate bentonite clay, particularly its sodium and calcium forms [129,130]. A diet with 227 g bentonite/cow/day could diminish AFM1 content in milk by 60.4% [131]. The addition of clay—including vermiculite, nontronite, and montmorillonite—to cow feed in 0.5, 1, and 2% concentration could reduce AFM1 excretion by 25, 18, 41%, respectively [127]. Activated carbon and glucomannan are very useful in lowering the AFM1 content of milk without changing milk composition [132]. Activated carbon mixed with hydrated sodium calcium aluminosilicate (HSCAS) mitigates the conversion of AFB1 to AFM1 by 36 to 50% [130]. In an in vitro study, Muhammad and Farhat, (2018) and Zahoor and Khan (2018) claimed that magnetic carbon particles prepared from bagasse, containing nanocrystalline iron oxide are used to detoxicate poultry feed as an alternative of the currently used powdered activated carbon adsorbent [133]. The mixture of activated carbon and HSCAS adsorbent could partially alleviate the negative effects of aflatoxin on blood profiles, growth performance, and hepatic gene expression in broilers [134]. The aluminosilicate mineral zeolite has a large internal surface, and high cation exchange capacity, therefore, can bind polar molecules [135]. Organically modified zeolites are more efficient adsorbents than the original natural minerals [136]. Diatomite (a sedimentary silica rock mineral) and sepiolite (a soft white clay mineral) have also been studied over the last few years and have proved to be effective adsorbents as well owing to their large surface [135].

Microfibers derive from cereals and legumes and contain mainly cellulose, hemicellulose, and lignin. Microfibers and bio-sorbents can adsorb mycotoxins favorably in the digestive tract and can be excreted easily with feces [137].

Many synthetic polymers, such as cholestyramine, divinylbenzene-styrene, and polyvinylpyrrolidone, have been shown to bind mycotoxins effectively [138]. More recently, Arak et al. (2019) have synthesized polymers based on methacrylic acid, and a macroporous molecularly imprinted polymer, TMU95, was efficient in binding AFB1 in the feed of ducklings. It also had beneficial effects on the growth of the livestock and alleviated the harmful physiological effects of the mycotoxin [139]. A new adsorbent was developed for the selective removal of AFM1 content of raw milk using a molecularly imprinted polymer, which is coated on the surface of the stainless-steel plate [140].

## 3. Potentials and Challenges of Upscaling Experimental Detoxifying Methods

The available physical and chemical AF mitigation and detoxification methods influence not only the toxin concentrations of feed and food, but have—at least in several cases—significant effects on nutritional status and food safety (Table 5). The advantage of most physical methods such as radiation, high-pressure treatments, and pulsed electric field lies is their advantageous toxicological safety properties [141,142]. However, meanwhile, thermal treatments, sorting, and dehulling may decrease other food risks including microbiological contaminations, pesticide residues, and toxic elements [143,144,145], they can also result in significant losses in nutrients [91,143]. Therefore, holistic approaches should be used to test the toxicological and nutritional consequences of treatments and the effects of sample matrices, since the efficiencies of physical methods are strongly dependent on the materials to be decontaminated and the application conditions [34,41,49,58]. Modern mathematical methods are available for multi-purpose optimization of processing parameters, taking into account the relationships existing between structure, composition, shelf-life, and safety [146,147]. All these evaluations should rely on a sufficient amount of high-quality data coming from carefully planned and reliable research. Furthermore, although a tested treatment may exhibit a promising mitigation effect in a laboratory experiment, numerous additional studies are likely to be performed when it is scaled up to a pilot or manufacturing scales to reveal limitations and impracticality and demonstrate the industrial applicability [148,149,150]. This means that the transition of a new mycotoxin decontamination technology from laboratory to manufacturing scale always needs a number of additional quality tests to be performed until a reliable and stable technological setup has been reached, which provides the consumers with the expected quality and safety of the food product [151].

## 4. Conclusions and Future Trends

Physical and chemical AF mitigation methods have been reviewed in this paper. Besides the widely used pre-harvest biocontrol methods, more recent research work performed in this field focuses more on the development and improvement of post-harvest processes as well including microwave heating, gamma irradiation, pulsed light and UV light, cold plasma, inorganic and organic acids, alkalis, gases, and mycotoxin-binding absorbents. The combination of these methods is expected to increase the efficiency of AF decontamination, which is also influenced by the nature of the products and the level of their AF contamination. One of the main future challenges is to develop new procedures that may achieve comparable detoxification efficiencies in a broad spectrum of feed and food matrices, because there are no such general all-purpose decontamination methods, which could be broadly employed. Notably, the novel emerging decontamination technologies should not change the physical–chemical properties of the treated feed and food products significantly, and no toxic residues of the mycotoxins should be left over in the decontaminated products. Future research should focus on the elaboration of these novel technologies and their extensive testing in as versatile feed and food matrices as possible.

## Figures and Tables

**Table 1:** Intervention levels to reduce aflatoxin hazard in the feed and food chain.

Level 1: Prevention	Level 2: Decontamination	Level 3: Detoxification
Biological control in field Good agricultural practice Good storage practice Good manufacturing practice	Removal Sorting Classification Absorption Filtration	Radiation and light treatments Thermal and pressure treatments Nonthermal plasma treatments Chemical agents

**Table 2:** Physical methods available for aflatoxin reduction in feed and food samples.

Principle	Method	Commodity	Reducing Efficiency
Removal	Sorting by size and density	Brazil nuts [31], Corn [32]	Only small nuts contained AFB1 98%
	Washing	Corn [28,33,34]	90–97%
	Color classification using UV fluorescent and multispectral analysis	Corn [35,36],	81–83%
	Removal of external grain parts (dehulling, polishing)	Corn [28,33,34,37], Rice [38]	<92% 88–92%
	Carbon filtration	AFs spiked liquid coffee samples [39]	74–79%
Reduction, Destruction	Thermal treatment	Wheat [40], Soybean [41], Peanuts [42], Pistachio [43]	50–90% 42–81% 57–80% 93%
	High moisture thermal treatment (roasting, extrusion, cooking, High-pressure cooking, instant catapult steam explosion)	Maize [44], Rice [45,46], Corn starch [47], Corn stalk [48]	51–85% 25–88% 75–87% 100%
	High hydrostatic pressure	Spiked grape juice [49], water [49]	14–29% 61–87%
	UV light, near-infrared radiation	Milk [50], Rice [51], Peanuts [52]	65–100% <99% 14–17%
	Gamma irradiation	Mixed poultry feed [53], Corn [54,55,56], Wheat [54], Rice [54], Soybean [57], Peanuts [58]	43% 15–90% 22–69% 27–65% 62–76% 20–43%
	Pulsed light treatment	Rice kernel and bran [59]	39–90%
	Pulsed electric field	Potato dextrose agar: [60,61], Sesame seed [62], Spiked grape juice [49]	79–96% 86.9–98.7% 24–82%
	Ultrasound	Corn flour [63]	11%
	Cold or nonthermal plasma treatment	Corn [32], Hazelnuts [64,65], Peanuts [66], Spiked food samples [67]	62–82 21–50% 23–38% 45–56%
	Electrolyzed water	Peanuts [68,69], Olive oil [69]	85–90% <99%

**Table 3:** Chemical methods available for aflatoxin reduction in feed and food samples.

Method	Commodity	Reducing Efficiency
Use of organic and inorganic acids (e.g., citric, lactic, tartaric, propionic, and hydrochloric acids)	Grains, mixed feed, black pepper, distillers’ grains, and condensed distillers’ solubles [87,88,89,90,92,94,95]	<92%
Ammoniation	Milk [96]	79–90%
Redox-active enzymes	AFs spiked liquid coffee samples [97]	<96%
Ozone treatment	Corn [98,99] Wheat [100] Pistachios [101] Poultry feed [102]	79–95% 85–95% 13% 86%

**Table 4:** Comparison of the flatoxin-binding efficiency of different absorbents.

Name of Binder	Concentration	Binding Efficiency	References
Activated carbon	1% suspension	>99.5%	[124]
Calcium bentonite	1% suspension	98.5%	[124]
Diatomite	50 mg/2 µg/mL AFB_1_	90–95%	[125]
Esterified glucomannan	1% suspension	96.6%	[124]
Hydrated sodium calcium aluminosilicate (HSCAS)	100 mg/2 µg/mL AFB_1_	98–100%	[126]
Vermiculite, nontronite, and montmorillonite	2% of feed	41%	[127]
Zeolite	82 mg/0.821 µg/mL AFB_1_	80%	[128]

**Table 5:** Nutritional and food safety effects of physical and chemical processes in feed and food.

Method	Principle	Method	Effect
		Washing	Changes in enzyme activity [152]
		Removal of external grain parts (dehulling, polishing	Losses in nutritional value (e.g., fibers, minerals vitamins) [143] Removal of contaminants (pesticide residues, toxic elements, microbes, other toxins) [143,144]
		Carbon filtration	Reduction in organic micropollutants [153]
	Reduction, destruction	Thermal treatment, high moisture thermal treatment (roasting, extrusion, cooking, high-pressure cooking, instant catapult steam explosion)	Losses in nutritional value (e.g., proteins, bioactive compounds) [91] Inactivation of microorganisms [145]
		High hydrostatic pressure	Inactivation of microorganisms and enzymes [142] Retention of organoleptic and nutritional properties [49,142]
		UV light, near-infrared radiation	Reduction in allergenicity of food proteins [154]
		Gamma irradiation	Reduce the allergenicity of food proteins, denature and agglomerate the proteins [155] Lipid and vitamin oxidization [151]
		Pulsed electric field	Inactivation of enzymes and microorganisms, safe for humans, because no dangerous chemical reactions have been detected [141] Low effect on nutritional and organoleptic properties [49] Structure of macromolecules (e.g., starch, protein) changes [156]
		Ultrasound	Reduce the allergenicity of food proteins [154] High-frequency low-power ultrasound has minimal physical and/or chemical effects on food constituents [157,158]
		Cold or nonthermal plasma treatment	Inactivation of microorganisms [159,160] No effect on nutritional properties [161] Improve technological properties [162]
		Electrolyzed water	Reduce the natural microbiota, no effect on nutritional properties [163]
Chemical		Liquid chemical agents (acids)	Changes in nutrient status and sensory properties, food safety concerns [10,164]
		Enzymes	Enzyme specific effects on quality, reduction in safety risks [165]
		Gaseous chemicals (ammonia, ozone)	Reduction in organic micropollutants [153] No hazard on treated materials [163] Oxidization of lipids and phenolic compounds [151]

## Data Availability

Not applicable.

## References

[1] Peles F., Sipos P., Győri Z., Pfliegler W.P., Giacometti F., Serraino A., Pagliuca G., Gazzotti T., Pócsi I. (2019). Adverse effects, transformation, and channeling of aflatoxins into food raw materials in livestock. Front. Microbiol..

[2] Ráduly Z., Szabó L., Madar A., Pócsi I., Csernoch L. (2019). Toxicological and medical aspects of Aspergillus-derived mycotoxins entering the feed and food chain. Front. Microbiol..

[3] Jalili M. (2015). A review on aflatoxins reduction in food. Iran. J. Health Saf. Environ..

[4] Frisvad J.C., Hubka V., Ezekiel C.N., Hong S.B., Nováková A., Chen A.J., Arzanlou M., Larsen T.O., Sklenář F., Mahakarnchanakul W. (2019). Taxonomy of Aspergillus section Flavi and their production of aflatoxins, ochratoxins and other mycotoxins. Stud. Mycol..

[5] Lizárraga-Paulín E.G., Miranda-Castro S.P., Moreno-Martínez E., Torres-Pacheco I., Lara-Sagahón A.V., Razzaghi-Abyaneh M. (2013). Novel methods for preventing and controlling aflatoxins in food: A worldwide daily challenge. Aflatoxins—Recent Advances and Future Prospects.

[6] Caceres I., El Khoury R., Bailly S., Oswald I.P., Puel O., Bailly J.D. (2017). Piperine inhibits aflatoxin B1 production in Aspergillus flavus by modulating fungal oxidative stress response. Fungal Genet. Biol..

[7] Reverberi M., Punelli F., Scarpari M., Camera E., Zjalic S., Ricelli A., Fanelli C., Fabbri A.A. (2010). Lipoperoxidation affects ochratoxin A biosynthesis in Aspergillus ochraceus and its interaction with wheat seeds. Appl. Genet. Mol. Biotechnol..

[8] Hong S.Y., Roze L.V., Wee J., Linz J.E. (2013). Evidence that a transcription factor regulatory network coordinates oxidative stress response and secondary metabolism in Aspergilli. Microbiologyopen.

[9] Pfliegler V., Pócsi I., Győri Z., Pusztahelyi T. (2020). The Aspergilli and their mycotoxins: Metabolic interactions with plants and the soil biota. Front. Microbiol..

[10] Peles F., Sipos P., Kovács S., Győri Z., Pócsi I., Pusztahelyi T. (2021). Biological Control and Mitigation of Aflatoxin Contamination in Commodities. Toxins.

[11] Torres A.M., Barros G.G., Palacios S.A., Chulze S.N., Battilani P. (2014). Review on pre- and post-harvest management of peanuts to minimize aflatoxin contamination. Food Res. Int..

[12] Assaf J.C., Khoury A., Chokr A., Louka N., Atoui A. (2019). A novel method for elimination of aflatoxin M1 in milk using Lactobacillus rhamnosus GG biofilm. Int. J. Dairy Technol..

[13] Assaf J.C., Nahle S., Chokr A., Louka N., Atoui A., El Khoury A. (2019). Assorted methods for decontamination of aflatoxin M1 in milk using microbial adsorbents. Toxins.

[14] Norlia M., Jinap S., Nor-Khaizura M., Radu S., Samsudin N., Azri F.A. (2019). Aspergillus section Flavi and aflatoxins: Occurrence, detection, and identification in raw peanuts and peanut-based products along the supply chain. Front. Microbiol..

[15] Serraino A., Bonilauri P., Kerekes K., Farkas Z., Giacometti F., Canever A., Zambrini A.V., Ambrus Á. (2019). Occurrence of aflatoxin M1 in raw milk marketed in Italy: Exposure assessment and risk characterization. Front. Microbiol..

[16] Tabata S. (1998). Aflatoxin contamination in foods and foodstuffs. Mycotoxins.

[17] Arab M., Sohrabvandi S., Mortazavian A.M., Mohammadi R., Rezaei Tavirani M. (2012). Reduction of aflatoxin in fermented milks during production and storage. Toxin Rev..

[18] Sharifzadeh A., Ghasemi-Dehkordi P., Foroughi M., Mardanpour-Shahrekordi E., Ramazie S. (2017). Aflatoxin M1 contamination levels in cheeses sold in Isfahan Province, Iran. Osong Public Health Res. Perspect..

[19] Shigute T., Washe A.P. (2018). Reduction of aflatoxin M1 levels during Ethiopian traditional fermented milk (Ergo) production. J. Food Qual..

[20] Maleki F., Abdi S., Davodian E., Haghani K., Bakhtiyari S. (2015). Exposure of infants to aflatoxin M1 from mother’s breast milk in Ilam, Western Iran. Osong Public Health Res. Perspect..

[21] Warth B., Braun D., Ezekiel C.N., Turner P.C., Degen G.H., Marko D. (2016). Biomonitoring of mycotoxins in human breast milk: Current state and future perspectives. Chem. Res. Toxicol..

[22] Fisher W.J., Schilter B., Tritsher A.M., Stadler R.H., Fuquay J.W., Fox P.F., McSweeney P.L.H. (2011). Environmental contaminants. Contaminants of milk and dairy products. Encyclopedia of Dariry Science.

[23] Bianchini A., Bullerman L.B., Appell M., Kendra D.F., Trucksess M.W. (2009). Biological Control of Molds and Mycotoxins in Foods. Mycotoxin Prevention and Control in Agriculture.

[24] Tian F., Chun H.S., Abdulra’uf L. (2017). Natural products for preventing and controlling aflatoxin contamination of food. Aflatoxin-Control, Analysis, Detection and Health Risks.

[25] Nagy R., Máthé E., Csapó J., Sipos P. (2021). Modifying Effects of Physical Processes on Starch and Dietary Fiber Content of Foodstuffs. Processes.

[26] Rustom I.Y.S. (1997). Aflatoxin in food and feed: Occurrence, legislation and inactivation by physical methods. Food Chem..

[27] Negash D. (2018). A review of aflatoxin: Occurrence, prevention, and gaps in both food and feed safety. J. Nutr. Health Food Eng..

[28] Matumba L., Van Poucke C., Ediage E.N., Jacobs B., De Saeger S. (2015). Effectiveness of hand sorting, flotation/washing, dehulling and combinations thereof on the decontamination of mycotoxin-contaminated white maize. Food Addit. Contam. Part A.

[29] Benkerroum N. (2020). Aflatoxins: Production, structure, health issues and incidence in Southeast Asian and Sub-Saharan African Countries. Int. J. Environ. Res. Public Health.

[30] Peng Z., Chen L., Zhu Y., Huang Y., Hu X., Wu Q., Nüssler A.K., Liu L., Yang W. (2018). Current major degradation methods for aflatoxins: A review. Trends Food Sci. Tech..

[31] De Mello F.R., Scussel V.M. (2007). Characteristics of in-shell Brazil nuts and their relationship to aflatoxin contamination: Criteria for sorting. J. Agric. Food Chem..

[32] Shi H., Ileleji K., Stroshine R.L., Keener K., Jensen J.L. (2017). Reduction of aflatoxin in corn by high voltage atmospheric cold plasma. Food Bioproc. Tech..

[33] Fandohan P., Zoumenou D., Hounhouigan D.J., Marasas W.F.O., Wingfield M.J., Hell K. (2005). Fate of aflatoxins and fumonisins during the processing of maize into food products in Benin. Int. J. Food Microbiol..

[34] Mutungi C., Lamuka P., Arimi S., Gathumbi J., Onyango C. (2008). The fate of aflatoxins during processing of maize into muthokoi – A traditional Kenyan food. Food Control.

[35] Pearson T.C., Wicklow D.T., Pasikatan M.C. (2004). Reduction of aflatoxin and fumonisin contamination in yellow corn by high-speed dual-wavelength sorting. Cher. Chem..

[36] Stasiewicz M.J., OFalade T.D., Mutuma M., Mutiga S.K., Harvey J.J.W., Fox G., Pearson T.C., Muthomi J.W., Nelson R.J. (2017). Multi-spectral kernel sorting to reduce aflatoxins and fumonisins in Kenyan maize. Food Control.

[37] Siwela A.H., Siwela M., Matindi G., Dube S., Nziramasanga N. (2005). Decontamination of aflatoxin-contaminated maize by dehulling. J. Sci. Food Agric..

[38] Castells M., Ramos A.J., Sanchis V., Marín S. (2007). Distribution of total aflatoxins in milled fractions of hulled rice. J. Agric. Food Chem..

[39] Azam K., Akhtar S., Gong Y.Y., Routledge M.N., Ismail A., Oliveira C.A.F., Iqbal S.Z., Ali H. (2021). Evaluation of the impact of activated carbon-based filtration system on the concentration of aflatoxins and selected heavy metals in roasted coffee. Food Control.

[40] Hwang J.-H., Lee K.-G. (2006). Reduction of aflatoxin B1 contamination in wheat by various cooking treatments. Food Chem..

[41] Lee J., Her J.-Y., Lee K.-G. (2015). Reduction of aflatoxins (B1, B2, G1, and G2) in soybean-based model systems. Food Chem..

[42] Arzandeh S., Jinap S. (2011). Effect of initial aflatoxin concentration, heating time and roasting temperature on aflatoxin reduction in contaminated peanuts and process optimisation using response surface modelling. Int. J. Food Sci. Technol..

[43] Rastegar H., Shoeibi S., Yazdanpanah H., Amirahmadi M., Khaneghah A.M., Campagnollo F.B., de Souza Sant’Ana A. (2017). Removal of aflatoxin B1 by roasting with lemon juice and/or citric acid in contaminated pistachio nuts. Food Control.

[44] Mtega M.M., Mgina C.A., Kaale E., Sempombe S., Kilulya K.F. (2020). Occurrence of Aflatoxins in Maize and Maize Products from Selected Locations of Tanzania and the Effects of Cooking Preparation Processes on Toxin Levels. Tanz. J. Sci..

[45] Sani A.M., Azizi E.G., Salehi E.A., Rahimi K. (2014). Reduction of aflatoxin in rice by different cooking methods. Toxicol. Ind. Health.

[46] Park J.W., Kim Y.B. (2006). Effect of pressure cooking on aflatoxin B1 in rice. J. Agric. Food Chem..

[47] Massarolo K.C., Mendoza J.R., Verma T., Kupski L., Badiale-Furlong E., Bianchini A. (2021). 2021: Fate of aflatoxins in cornmeal during single-screw extrusion: A bioaccessibility approach. LWT.

[48] Xie H., Li Z., Wang Z., Mao G., Zhang H., Wang F., Chen H., Yang S., Tsang Y.F., Lam S.S. (2020). Instant Catapult Steam Explosion: A rapid technique for detoxification of aflatoxin-contaminated biomass for sustainable utilization as animal feed. J. Clean. Prod..

[49] Pallarés N., Berrada H., Tolosa J., Ferrer E. (2021). Effect of high hydrostatic pressure (HPP) and pulsed electric field (PEF) technologies on reduction of aflatoxins in fruit juices. LWT.

[50] Hassan F.F., Hussein H.Z. (2017). Detection of aflatoxin M1 in pasteurized canned milk and using of UV radiation for detoxification. Int. J. Adv. Chem. Eng. Biol. Sci..

[51] Ferreira C.D., Lang G.H., da Silva Lindemann I., da Silva Timm N., Hoffmann J.F., Ziegler V., de Oliveira M. (2021). Postharvest UV-C irradiation for fungal control and reduction of mycotoxins in brown, black, and red rice during long-term storage. Food Chem..

[52] Shen M.H., Singh R. (2021). Effect of rotating peanuts on aflatoxin detoxification by ultraviolet C light and irradiation uniformity evaluated by AgCl-based dosimeter. Food Control.

[53] Herzallah S., Alshawabkeh K., Fataftah A.A.L. (2008). Aflatoxin decontamination of artificially contaminated feeds by sunlight, γ-radiation, and microwave heating. J. Appl. Poult. Res..

[54] Mohamed N.F., El-Dine R.S.S., Kot M.A.M., Saber A. (2015). Assessing the possible effect of gamma irradiation on the reduction of aflatoxin B1, and on the moisture content in some cereal grains. Am. J. Biomed. Sci..

[55] Markov K., Mihaljević B., Domijan A.-M., Pleadin J., Delaš F., Frece J. (2015). Inactivation of aflatoxigenic fungi and the reduction of aflatoxin B1 in vitro and in situ using gamma irradiation. Food Control.

[56] Serra M.S., Pulles M.B., Mayanquer F.T., Vallejo M.C., Rosero M.I., Ortega J.M., Naranjo L.N. (2018). Evaluation of the use of gamma radiation for reduction of aflatoxin B1 in corn (*Zea mays*) used in the production of feed for broiler chickens. J. Agric. Chem. Environ..

[57] Zhang Z.S., Xie Q.F., Che L.M. (2018). Effects of gamma irradiation on aflatoxin B 1 levels in soybean and on the properties of soybean and soybean oil. Appl. Radiat. Isot..

[58] Patil H., Shah N.G., Hajare S.N., Gautam S., Kumar G. (2019). Combination of microwave and gamma irradiation for reduction of aflatoxin B1 and microbiological contamination in peanuts (*Arachis hypogaea* L.). World Mycotoxin J..

[59] Wang B., Mahoney N.E., Pan Z., Khir R., Wu B., Ma H., Zhao L. (2016). Effectiveness of pulsed light treatment for degradation and detoxification of aflatoxin B1 and B2 in rough rice and rice bran. Food Control.

[60] Vijayalakshmi S., Nadanasabhapathi S., Kumar R., Kumar S., Reddy R. (2017). Effect of combination processing on aflatoxin reduction: Process optimization by response surface methodology. J. Food Process. Preserv..

[61] Vijayalakshmi S., Nadanasabhapathi S., Kumar R., Kumar S.S. (2018). Effect of pH and pulsed electric field process parameters on the aflatoxin reduction in model system using response surface methodology. J. Food Sci. Technol..

[62] Bulut N., Atmaca B., Evrendilek G.A., Uzuner S. (2020). Potential of pulsed electric field to control Aspergillus parasiticus, aflatoxin and mutagenicity levels: Sesame seed quality. J. Food Saf..

[63] Liu Y., Li M., Bai F., Bian K. (2019). Effects of pulsed ultrasound at 20 kHz on the sonochemical degradation of mycotoxins. World Mycotoxin J..

[64] Basaran P., Basaran-Akgul N., Oksuz L. (2008). Elimination of Aspergillus parasiticus from nut surface with low pressure cold plasma (LPCP) treatment. Food Microbiol..

[65] Sen Y., Onal-Ulusoy B., Mutlu M. (2019). Detoxification of hazelnuts by different cold plasmas and gamma irradiation treatments. Innov. Food Sci. Emerg. Technol..

[66] Iqdiam B.M., Abuagela M.O., Boz Z., Marshall S.M., Goodrich-Schneider R., Sims C.A., Marshall M.R., MacIntosh A.J., Welt B.A. (2020). Effects of atmospheric pressure plasma jet treatment on aflatoxin level, physiochemical quality, and sensory attributes of peanuts. J. Food Process. Preserv..

[67] Puligundla P., Lee T., Mok C. (2020). 2020: Effect of corona discharge plasma jet treatment on the degradation of aflatoxin B1 on glass slides and in spiked food commodities. LWT.

[68] Pankaj S.K., Shi H., Keener K.M. (2018). A review of novel physical and chemical decontamination technologies for aflatoxin in food. Trends Food Sci. Technol..

[69] Yang Q., Long X.D. (2019). Decontamination of aflatoxin B1. Aflatoxin B1 Occurrence, Detection and Toxicological Effects.

[70] Ryu D., Bianchini A., Bullerman L.B. (2008). Effects of processing on mycotoxins. Stewart Postharvest Rev..

[71] Wgiorgis G.A., Yildiz F. (2019). Review on high-pressure processing of foods. Cogent Food Agric..

[72] Pasikatan M.C., Dowell F.E. (2001). Sorting systems based on optical methods for detecting and removing seeds infested internally by insects or fungi: A review. Appl. Spectrosc. Rev..

[73] Tao F., Yao H., Hruska Z., Burger L.W., Rajasekaran K., Bhatnagar D. (2018). Recent development of optical methods in rapid and non-destructive detection of aflatoxin and fungal contamination in agricultural products. Trends Analyt. Chem..

[74] Eisa N.A., Ali F.M., El-Habbaa G.M., Abdel-Reheem S.K., Abou-El-Ella M.F. (2020). Pulsed electric field technology for checking aflatoxin production in cultures and corn grains. Egypt. J. Phytopathol..

[75] Vanga S.K., Wang J., Orsat V., Raghavan V. (2020). Effect of pulsed ultrasound, a green food processing technique, on the secondary structure and in-vitro digestibility of almond milk protein. Food Res. Int..

[76] Kumar V.V. (2018). Aflatoxins: Properties, toxicity, and detoxification. Nutr. Food Sci. Int. J..

[77] Patras A., Julakanti S., Yannam S., Bansode R.R., Burns M., Vergne M.J. (2017). Effect of UV irradiation on aflatoxin reduction: A cytotoxicity evaluation study using human hepatoma cell line. Mycotoxin Res..

[78] Moreau M., Lescure G., Agoulon A., Svinareff P., Orange N., Feuilloley M. (2013). Application of the pulsed light technology to mycotoxin degradation and inactivation. J. Appl. Toxicol..

[79] Woldemariam H.W., Kießling M., Emire S.A., Teshome P.G., Töpfl S., Aganovic K. (2021). Influence of electron beam treatment on naturally contaminated red pepper (*Capsicum annuum* L.) powder: Kinetics of microbial inactivation and physicochemical quality changes. Innov. Food Sci. Emerg. Technol..

[80] Misra N.N., Yadav B., Roopesh M.S., Jo C. (2019). Cold plasma for effective fungal and mycotoxin control in foods: Mechanisms, inactivation effects, and applications. Compr. Rev. Food Sci. Food Saf..

[81] Hojnik N., Cvelbar U., Tavcar-Kalcher G., Walsh J.L., Križaj I. (2017). Mycotoxin decontamination of food: Cold atmospheric pressure plasma versus “classic” decontamination. Toxins.

[82] Hojnik N., Modic M., Walsh J.L., Zigon D., Javornik U., Plavec J., Zegura B., Filipic M., Cvelbar U. (2021). 2021: Unravelling the pathways of air plasma induced aflatoxin B1 degradation and detoxification. J. Hazard. Mater..

[83] Escobedo-González R., Méndez-Albores A., Villarreal-Barajas T., Aceves-Hernández J., Miranda-Ruvalcaba R., Nicolás-Vázquez I. (2016). A theoretical study of 8-chloro-9-hydroxy-aflatoxin B1, the conversion product of aflatoxin B1 by neutral electrolyzed water. Toxins.

[84] Awuah S.K., Kumah P., Tandoh P.K. (2019). Effect of packaging materials on insect mortality and aflatoxin contamination in stored maize under different conditions. J. Exp. Agric. Int..

[85] Nwaubani S.I., Otitodun G.O., Ajao S.K., Opit G.P., Ala A.A., Omobowale M.O., Ogwumike J.C., Abel G.I., Ogundare M.O., Braimah J.A. (2020). Assessing efficacies of insect pest management methods for stored bagged maize preservation in storehouses located in Nigerian markets. J. Stored Prod. Res..

[86] Masters W.A., Guevara A.G. (2018). Willingness to Pay for Hermetic Grain Storage Bags in MALAWI. https://ageconsearch.umn.edu/record/27729510.22004/ag.econ.277295.

[87] Rushing B., Selim M.I. (2019). Aflatoxin B1: A review on metabolism, toxicity, occurrence in food, occupational exposure, and detoxification methods. Food Chem. Toxicol..

[88] Jubeen F., Sher F., Hazafa A., Zafar F., Ameen M., Rasheed T. (2020). Evaluation and detoxification of aflatoxins in ground and tree nuts using food grade organic acids. Biocatal. Agric. Biotechnol..

[89] Méndez-Albores A., Del Río-García J.C., Moreno-Martínez E. (2007). Decontamination of aflatoxin duckling feed with aqueous citric acid treatment. Anim. Feed Sci. Technol..

[90] Méndez-Albores A., Martínez-Bustos F., Gaytán-Martínez M., Moreno-Martínez E. (2008). Effect of lactic and citric acid on the stability of B-aflatoxins in extrusion cooked sorghum. Lett. Appl. Microbiol..

[91] Čolović R., Puvača N., Cheli F., Avantaggiato G., Greco D., Đuragić O., Kos J., Pinotti L. (2019). Decontamination of mycotoxin-contaminated feedstuffs and compound feed. Toxins.

[92] Altug T., Yousef A.E., Marth E.H. (1990). Degradation of aflatoxin B1 in dried figs by sodium bisulfite with or without heat, ultraviolet energy or hydrogen peroxide. J. Food Prot..

[93] Shi H., Stroshine R., Ileleji K. (2017). Determination of the relative effectiveness of four food additives in degrading aflatoxin in distillers wet grains and condensed distillers solubles. J. Food Prot..

[94] Jalili M., Jinap S. (2012). Role of sodium hydrosulphite and pressure on the reduction of aflatoxins and ochratoxin A in black pepper. Food Control.

[95] Jalili M., Jinap S., Son R. (2011). The effect of chemical treatment on reduction of aflatoxins and ochratoxin A in black and white pepper during washing. Food Addit. Contam. Part A Chem. Anal. Control Expo. Risk Assess..

[96] Phillips T.D. (1999). Dietary clay in the chemoprevention of aflatoxin-induced disease. Toxicol. Sci..

[97] Loi M., Renaud J.B., Rosini E., Pollegioni L., Vignali E., Haidukowski M., Sumarah M.W., Logrieco A.F., Mul G. (2020). 2020: Enzymatic transformation of aflatoxin B1 by Rh_DypB peroxidase and characterization of the reaction products. Chemosphere.

[98] McKenzie K.S., Kubena L.F., Denvir A.J., Rogers T.D., Hitchens G.D., Bailey R.H., Harvey R.B., Buckley S.A., Phillips T.D. (1998). Aflatoxicosis in turkey poults is prevented by treatment of naturally contaminated corn with ozone generated by electrolysis. Poult. Sci..

[99] Luo X., Wang R., Wang L., Li Y., Bian Y., Chen Z. (2014). Effect of ozone treatment on aflatoxin B1 and safety evaluation of ozonized corn. Food Control.

[100] Savi G.D., Piacentini K.C., Scussel V.M. (2015). Ozone treatment efficiency in Aspergillus and Penicillium growth inhibition and mycotoxin degradation of stored wheat grains (*Triticum aestivum* L.). J. Food Process. Preserv..

[101] Isikber A.A., Athanassiou C.G. (2015). The use of ozone gas for the control of insects and micro-organisms in stored products. J. Stored Prod. Res..

[102] Torlak E., Akata I., Erci F., Uncu A.T. (2016). Use of gaseous ozone to reduce aflatoxin B1 and microorganisms in poultry feed. J. Stored Prod. Res..

[103] Tiwari B.W., Brennan C.S., Curran T., Gallagher E., Cullen P.J., O’ Donnell C.P. (2010). Application of ozone in grain processing. J. Cereal Sci..

[104] Zhu F. (2018). Effect of ozone treatment on the quality of grain products. Food Chem..

[105] Ismail A., Gonçalves B.L., de Neeff D.V., Ponzilacqua B., Coppa C.F.S.C., Hintzsche H., Sajid M., Cruz A.G., Corassin C.H., Oliveira C.F.A. (2018). Aflatoxin in foodstuffs: Occurrence and recent advances in decontamination. Food Res. Int..

[106] Park D.L., Price W.D. (2001). Reduction of aflatoxin hazards using ammoniation. Rev. Environ. Contam. Toxicol..

[107] Yu Y., Shi J., Xie B., He Y., Qin Y., Wang D., Shi H., Ke Y., Sun Q. (2020). Detoxification of aflatoxin B1 in corn by chlorine dioxide gas. Food Chem..

[108] Zahoor M., Khan F.A. (2016). 2012: Aflatoxin B1 detoxification by magnetic carbon nanostructures prepared from maize straw. Desalin. Water Treat..

[109] Ji J., Xie W. (2020). 2020: Detoxification of Aflatoxin B1 by magnetic graphene composite adsorbents from contaminated oils. J. Hazard. Mater..

[110] Ji  J., Xie W. (2021). 2021: Removal of aflatoxin B1 from contaminated peanut oils using magnetic attapulgite. Food Chem..

[111] Sun S., Zhao R., Xie Y., Liu Y. (2021). Reduction of aflatoxin B1 by magnetic graphene oxide/TiO_2_ nanocomposite and its effect on quality of corn oil. Food Chem..

[112] Udomkun P., Njukwe E., Rai M., Abd-Elsalam K. (2020). Nanotechnological methods for aflatoxin control. Nanomycotoxicology: Treating Mycotoxins in the Nano Way.

[113] Kujur A., Kumar A., Yadav A., Prakash B. (2020). Antifungal and aflatoxin B1 inhibitory efficacy of nanoencapsulated Pelargonium graveolens L. essential oil and its mode of action. LWT.

[114] Vila-Donat P., Marín S., Sanchis V., Ramos A.J. (2018). A review of the mycotoxin adsorbing agents, with an emphasis on their multi-binding capacity, for animal feed decontamination. Food Chem. Toxicol..

[115] Wielogórska E., MacDonald S., Elliott C.T. (2016). A review of the efficacy of mycotoxin detoxifying agents used in feed in light of changing global environment and legislation. World Mycotoxin J..

[116] Holanda D.M., Kim S.W. (2021). Mycotoxin occurrence, toxicity, and detoxifying agents in pig production with an emphasis on deoxynivalenol. Toxins.

[117] Hamza Z., El-Hashash M., Aly S., Hathout A., Soto E., Sabry B., Ostroff G. (2019). Preparation and characterization of yeast cell wall beta-glucan encapsulated humic acid nanoparticles as an enhanced aflatoxin B1 binder. Carbohydr. Polym..

[118] Nazarizadeh H., Pourreza J. (2019). Evaluation of three mycotoxin binders to prevent the adverse effects of aflatoxin B1 in growing broilers. J. Appl. Anim. Res..

[119] Solís-Cruz B., Hernández-Patlán D., Beyssac E., Latorre J.D., Hernandez-Velasco X., Merino-Guzman R., Tellez G., López-Arellano R. (2017). Evaluation of chitosan and cellulosic polymers as binding adsorbent materials to prevent aflatoxin B1, fumonisin B1, ochratoxin, trichothecene, deoxynivalenol, and zearalenone mycotoxicoses through an in vitro gastrointestinal model for poultry. Polymers.

[120] Chefchaou H., Mzabi A., Tanghort M., Moussa H., Chami N., Chami F., Remmal A. (2019). A comparative study of different mycotoxin adsorbents against DON, T2 toxin, aflatoxins and fumonisins production in maize flour. Livest. Res. Rural. Dev..

[121] Pappas A.C., Tsiplakou E., Tsitsigiannis D.I., Georgiadou M., Iliadi M.K., Sotirakoglou K., Zervas G. (2016). The role of bentonite binders in single or concomitant mycotoxin contamination of chicken diets. Br. Poult. Sci..

[122] Murugesan G.R., Ledoux D.R., Naehrer K., Berthiller F., Applegate T.J., Grenier B., Phillips T.D., Schatzmayr G. (2015). Prevalence and effects of mycotoxins on poultry health and performance, and recent development in mycotoxin counteracting strategies. Poult. Sci..

[123] Alvarado A.M., Zamora-Sanabria R., Granados-Chinchilla F., Abdulra’Uf L. (2017). A Focus on aflatoxins in feedstuffs: Levels of contamination, prevalence, control strategies, and impacts on animal health. Aflatoxin—Control, Analysis, Detection and Health Risks.

[124] Duarte D.E., Winston M.H., Brinton A.H., Lon W.W. (2003). Aflatoxin Binders I: In vitro binding assay for aflatoxin B1 by several potential sequestering agents. Mycopathologia.

[125] Bocarov-Stancic A., Adamovic M., Salma N., Bodroža-Solarov M., Vučković J., Pantić V. (2011). In vitro efficacy of mycotoxins adsorption by natural mineral adsorbents. Biotech. Anim. Husbandry..

[126] Li J., Suo D., Su X. (2010). Binding capacity for aflatoxin B1 by different adsorbents. Agric. Sci. China.

[127] Sulzburger S.A., Melnichenko S., Cardoso F.C. (2017). Effects of clay after an aflatoxin challenge on aflatoxin clearance, milk production, and metabolism of Holstein cows. J. Dairy Sci..

[128] Gallo A., Masoero F. (2010). In vitro models to evaluate the capacity of different sequestering agents to adsorb aflatoxins. Ital. J. Anim. Sci..

[129] Jaynes W., Zartman R., Hudnall W. (2007). Aflatoxin B1 adsorption by clays from water and corn meal. Appl. Clay Sci..

[130] Naeimipour F., Aghajani J., Kojuri S.A., Ayoubi S. (2018). Useful approaches for reducing aflatoxin M1 content in milk and dairy products. Biomed. Biotechnol. Res. J..

[131] Kissell L., Davidson S., Hopkins B.A., Smith G.W., Whitlow L.W. (2013). Effect of experimental feed additives on aflatoxin in milk of dairy cows fed aflatoxin-contaminated diets. J. Anim. Physiol. Anim. Nutr. (Berl.).

[132] Abdel-Wahhab M.A., Kholif A.M. (2008). Mycotoxins in animal feeds and prevention strategies: A review. Asian J. Anim. Sci..

[133] Zahoor M., Khan F.A. (2018). Adsorption of aflatoxin B1 on magnetic carbon nanocomposites prepared from bagasse. Arab. J. Chem..

[134] Liu J.B., Yan H.L., Cao S.C., Hu Y.D., Zhang H.F. (2020). Effects of absorbents on growth performance, blood profiles and liver gene expression in broilers fed diets naturally contaminated with aflatoxin. Asian-Australas. J. Anim. Sci..

[135] Di Gregorio M.C., de Neeff D.V., Jager A.V., Corassin C.H., de Pinho Carão A.C., de Albuquerque R., de Azevedo A.C., Oliveira C.A.F. (2014). Mineral adsorbents for prevention of mycotoxins in animal feeds. Toxin Rev..

[136] Baglieri A., Reyneri A., Gennari M., Negre M. (2013). Organically modified clays as binders of fumonisins in feedstocks. J. Environ. Sci. Health B.

[137] Aoudia N., Callu P., Grosjean F., Larondelle Y. (2009). Effectiveness of mycotoxin sequestration activity of micronized wheat fibres on distribution of ochratoxin A in plasma, liver and kidney of piglets fed a naturally contaminated diet. Food Chem. Toxicol..

[138] Avantaggiato G., Solfrizzo M., Visconti A. (2005). Recent advances on the use of adsorbent materials for detoxification of Fusarium mycotoxins. Food Addit. Contam..

[139] Arak H., Mohammad A.K.T., Mehdi H., Shaban R. (2019). The first in vivo application of synthetic polymers based on methacrylic acid as an aflatoxin sorbent in an animal model. Mycotoxin Res..

[140] Bodbodak S., Hesari J., Peighambardoust S.H., Mahkam M. (2018). Selective decontamination of aflatoxin M1 in milk by molecularly imprinted polymer coated on the surface of stainless steel plate. Int. J. Dairy Technol..

[141] Nowosad K., Sujka M., Pankiewicz U., Kowalski R. (2021). The application of PEF technology in food processing and human nutrition. J. Food Sci. Technol..

[142] Considine K.M., Kelly A.L., Fitzgerald G.F., Hill C., Sleator R.D. (2008). High-pressure processing—Effects on microbial food safety and food quality. FEMS Microbiol. Lett..

[143] Pedron T., Segura F.R., Paniz F.P., de Moura Souza F., dos Santos M.C., de Magalhães Júnior A.M., Batista B.L. (2019). Mitigation of arsenic in rice grains by polishing and washing: Evidencing the benefit and the cost. J. Cereal Sci..

[144] Román-Ochoa Y., Choque Delgado G.T., Tejada T.R., Yucra H.R., Durand A.E., Hamaker B.R. (2021). Heavy metal contamination and health risk assessment in grains and grain-based processed food in Arequipa region of Peru. Chemosphere.

[145] Den Besten H.M.W., Wells-Bennik M.H.J., Zwietering M.H. (2018). Natural Diversity in Heat Resistance of Bacteria and Bacterial Spores: Impact on Food Safety and Quality. Annu. Rev. Food Sci. Technol..

[146] Banga J.R., Balsa-Canto E., Moles C.G., Alonso A.A. (2003). Improving food processing using modern optimization methods. Trends Food Sci. Technol..

[147] Sevda S., Garlapati V.K., Singh A., Sevda S., Singh A. (2020). Role of mathematical and statistical modelling in food engineering. Mathematical and Statistical Applications in Food Engineering.

[148] Lonsane B.K., Saucedo-Castaneda G., Raimbault M., Roussos S., Viniegra-Gonzalez G., Ghildyal N.P., Ramakrishna M., Krishnaiah M.M. (1992). Scale-Up Strategies for Solid State Fermentation Systems. Process Biochem..

[149] Akoto E.Y., Klu Y.A.K., Lamptey M., Asibuo J.Y., Davis J., Phillips R., Jordan D., Rhoads J., Hoistington D., Chen J. (2017). Use of peanut meal as a model matrix to study the effect of composting on aflatoxin decontamination. World Mycotoxin J..

[150] Kramer B., Wunderlich J., Muranyi P. (2017). Recent findings in pulsed light disinfection. J. Appl. Microbiol..

[151] Mir S.A., Dar B.N., Shah M.A., Sofi S.A., Hamdani A.M., Oliveira C.A.F., Moosavi M.H., Khanegha A.M., Sant’Ana A.S. (2021). Application of new technologies in decontamination of mycotoxins in cereal grains: Challenges, and perspectives. Food Chem. Toxicol..

[152] De Almeida J.L., Pareyt B., Gerits L.R., Delcour J.A. (2014). Effect of wheat grain steaming and washing on lipase activity in whole grain flour. Cereal Chem..

[153] Reungoat J., Macova M., Escher B.I., Carswell S., Mueller J.F., Kellera J. (2010). Removal of micropollutants and reduction of biological activity in a full scale reclamation plant using ozonation and activated carbon filtration. Water Res..

[154] Lima F., Vieira K., Santos M., de Souza P.M., Diaz A.V., Garcia-Gimeno R.M. Effects of radiation technologies on food nutritional quality. Descriptive Food Science.

[155] Ahmed M.M., Abdalla I.G., Salih A.M., Hassan A.B. (2018). Effect of gamma radiation on storability and functional properties of sorghum grains (*Sorghum bicolor* L.). Food Sci. Nutr..

[156] Picart-Palmade L., Cunault C., Chevalier-Lucia D., Belleville M.P., Marchesseau S. (2019). Potentialities and Limits of Some Non-Thermal Technologies to Improve Sustainability of Food Processing. Front. Nutr..

[157] Ojha K.S., Tiwari B.K., O’Donnell C.P. (2018). Chapter Six—Effect of Ultrasound Technology on Food and Nutritional Quality. Adv. Food Nutr. Res..

[158] Gallo M., Ferrara L., Naviglio D. (2018). Application of ultrasound in food science and technology: A perspective. Foods.

[159] Dasan B.G., Yildirim T., Boyaci I.H. (2018). Surface decontamination of eggshells by using non-thermal atmospheric plasma. Int. J. Food Microbiol. Int. J. Food Microbiol..

[160] Misra N.N., Jo C. (2017). Applications of cold plasma technology for microbiological safety in meat industry. Trends Food Sci. Technol..

[161] Charoux C.M.G., Free L., Hinds L.M., Vijayaraghavan R.K., Daniels S., O’Donnell C.P., Tiwari B.K. (2019). Effect of non-thermal plasma technology on microbial inactivation and total phenolic content of model liquid food and black pepper grains. LWT.

[162] Thirumdas R., Saragapani C., Ajinkya M.T., Deshmukh R.R., Annapure U.S. (2016). Influence of low pressure cold plasma on cooking and textural properties of brown rice. Innov. Food Sci. Emerg. Technol..

[163] Zhang C., Zhang Y., Zhao Z., Liu W., Chen Y., Yang G., Xia X., Cao Y. (2019). The application of slightly acidic electrolyzed water in pea sprout production to ensure food safety, biological and nutritional quality of the sprout. Food Control.

[164] Udomkun P., Wiredu A.N., Nagle M., Müller J., Vanlauwe B., Bandyopadhyay R. (2017). Innovative technologies to manage aflatoxins in foods and feeds and the profitability of application—A review. Food Control.

[165] Zhang Y., He S., Simpson B.K. (2018). Enzymes in Food Bioprocessing—Novel food enzymes, applications, and related techniques. Curr. Opin. Food Sci..

